# Evaluating Elastographic Tendon Stiffness as a Predictor of Return to Work and Sports After Primary Rotator Cuff Repair

**DOI:** 10.1177/23259671241306761

**Published:** 2025-02-26

**Authors:** Alexander G. Maloof, Lisa Hackett, James Bilbrough, Christyon Hayek, George A.C. Murrell

**Affiliations:** * University of New South Wales, Sydney, New South Wales, Australia; † Orthopaedic Research Institute, Saint George Hospital, New South Wales, Australia; Investigation performed at the University of New South Wales, Sydney, Australia

**Keywords:** diagnostic ultrasound, imaging, return to sports, return to work, rotator cuff, rotator cuff repair, shoulder, shear wave elastography ultrasound, tendon stiffness

## Abstract

**Background:**

Despite many patients not returning to their preoperative levels of work and sports after primary rotator cuff repair, few studies have investigated the association between findings on postoperative imaging and patients returning to work and sports. Shear wave elastography ultrasound (SWEUS) is a recent technology that quantifies the stiffness of healing repaired cuff tendons.

**Hypothesis:**

Stiffer repaired cuff tendons that reflect improved healing would be associated with improved return to work and sports.

**Study Design:**

Cohort study; Level of evidence, 2.

**Methods:**

This was a prospective cohort study of 50 patients undergoing primary arthroscopic rotator cuff repair. Preoperatively, all patients completed a questionnaire ranking their level of sports and work activity on a 4-point Likert scale. At 8 days, 6 weeks, 12 weeks, 6 months, and 1 year postoperatively, patients reported their current levels of sports and work on the same scale and had SWEUS stiffness measurements taken at 3 points along their repaired tendons.

**Results:**

The elastographic stiffness of supraspinatus tendons at their repaired insertion sites increased by a mean of 22% over 12 months (*P* = .0001). Elevated supraspinatus tendon stiffness at 6 weeks and 12 weeks was associated with return to sports at 12 months (*r* = 0.46; *P* = .003 and *r* = 0.39; *P* = .01), and tendon stiffness at 12 weeks and 6 months was associated with return to work at 12 months (*r* = 0.49; *P* = .001 and *r* = 0.46; *P* = .003). Patients returning to sports (*r* = 0.46; *P* = .003) and work (*r* = 0.49; *P* = .001) were most strongly associated with SWEUS stiffness 12 weeks after cuff repair.

**Conclusion:**

The elastographic stiffness of a healing repaired supraspinatus tendon is moderately associated with improved return to work and sports 12 months after rotator cuff repair. Tendon stiffness at 12 weeks postrepair was the most critical timepoint in predicting both return to work and sports at 12 months postrepair.

More than 35% of patients are unable to return to previous levels of work and sports after rotator cuff (RCR) repair at 8 months.^8^ Predominantly presurgical demographic factors have been assessed for their association with the likelihood of patient return to work and sports outcomes. In a study on 63 patients who underwent arthroscopic rotator cuff repair, Imai et al^10^ found that female sex and engagement in heavy work preoperatively were associated with poorer recovery of working capability. Pichené-Houard et al^15^ conducted a study on 96 patients and found that perceived health before surgery and the physical demands of each patient's occupation were associated with an improved return to work trajectory after rotator cuff repair. Moreover, a meta-analysis by Altintas et al^2^ found that patients who participated in recreational sports were more likely to return to sports after cuff repair compared with those participating in competitive and overhead sports.

To date, no studies have assessed the relationship between the quality of the healing rotator cuff tendon after surgical repair and patient return to work and sports. A potential method to investigate the quality of a healing supraspinatus tendon is via shear wave elastography ultrasound (SWEUS)—a recent advancement in ultrasound technology that enables an ultrasound machine to quantify the stiffness of soft tissues, including rotator cuff tendons.^4,19^ Healthy and stiffer tendons have greater conduction velocity values (approaching 10 m/s) (shown in red in Figure 1).^1^ Tendinopathic tendons have a lower velocity (approaching 0.05 m/s) and softer and less stiff tissue^12,19^ (shown in blue in Figure 1).

**Figure 1.**
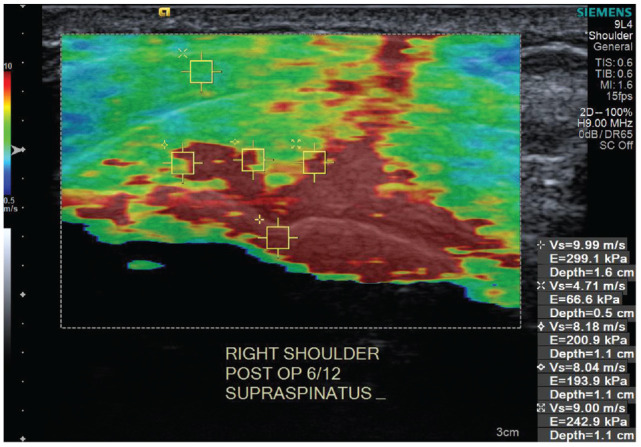
SWEUS *color elastogram* of the right supraspinatus tendon, which shows stiff tissue in red and soft tissue in blue (from Hackett et al^7^). SWEUS, shear wave elastography ultrasound.

SWEUS has been shown to be a reliable tool for diagnosing tendinopathy of the rotator cuff.^11^ Studies by Taljanovic et al^19^ and Hou et al^9^ noted that normal, healthy male supraspinatus tendons have an 8 to 9 m/s SWEUS velocity, while tendinopathic tendons, being those with reduced tendon integrity, have a SWEUS velocity of 5 to 7 m/s. Hou et al^9^ also evaluated the association of SWEUS velocity to the degree of supraspinatus tendinopathy and found the morphological grade of supraspinatus tendinopathy on B-mode ultrasound to be associated with decreased supraspinatus tendon stiffness (*P* < .003), demonstrating SWEUS capacity to identify tendons with decreased tensile properties. Hackett et al,^7^ at our institution, found SWEUS measurements to exhibit excellent intraobserver (intraclass correlation coefficient [ICC], 0.96) and moderate interobserver reliability (ICC, 0.45), similar to study findings by Baumer et al^3^ (ICC, 0.87).

To our knowledge, no study has compared postrotator cuff repair tendon stiffness and the level of postoperative patient work and sports activity. This study aimed to determine the following: (1) whether the supraspinatus tendon stiffness after rotator cuff repair is associated with an increased likelihood of returning to sports and work activity; (2) which timepoint, if any, has the strongest association between the stiffness of the repaired tendons and return to sports and work; and (3) whether SWEUS velocity measurements of the supraspinatus tendon change up to 12 months postoperatively. We hypothesized that healing supraspinatus tendons would become stiffer over time after repair. Since tendon stiffness after an initial recovery period has been associated with improved healing as time goes on after repair,^13^ we also hypothesized that there would be a positive association between the stiffness of the supraspinatus tendon after it has been repaired and the level of activity in sports and work to which patients return 12 months after repair. That is, patients whose repaired tendons are of greater stiffness, as measured by SWEUS postoperatively, would be more likely to return to a more active level of sports and work participation 12 months after rotator cuff repair.

## Methods

### Study Design

This was a prospective case series evaluating the association between the elastographic stiffness of repaired supraspinatus tendons and the level of work and sports to which participants return up to 12 months postoperatively.

### Ethics Approval

The local ethics committee approved this study to be conducted at the Orthopaedic Research Institute, Saint George Hospital, Sydney, Australia (SESLHD HREC Ethic: 2019/ETH09238).

### Cohort Study Design

#### Inclusion Criteria

The inclusion criteria were patients aged at least 18 years who underwent primary arthroscopic rotator cuff repair on an isolated tear in the supraspinatus tendon by the same surgeon (G.A.C.M.) and returned for their follow-up 8-day, 6-week, 12-week, 6-month, and 12-month postoperative appointments, where SWEUS was conducted on 3 points of the repaired supraspinatus tendon by the same experienced musculoskeletal sonographer (L.H.).

#### Exclusion Criteria

The exclusion criteria for this study were patients undergoing revision surgery, any repairs that involved concurrent shoulder or biceps procedures—such as synthetic patches, acromioplasty, calcific tendinitis debridement, distal clavicular resection or stabilization, partial repairs, significant muscle atrophy determined by B-mode ultrasound, glenohumeral arthritis (≥2 grade )—or patients with an avulsed fracture or isolated subscapularis tear. Any patients whose tendons retore after repair were excluded from further statistical analyses once the tear was identified.

#### Patient Recruitment

Patients undergoing primary arthroscopic rotator cuff repair by the same experienced shoulder surgeon upon being diagnosed with a supraspinatus tear were recruited during their preoperative appointment. Patient age, sex, and levels of preoperative work and sports were collected via a standard questionnaire after diagnosis of a rotator cuff tear on B-mode ultrasound by the same experienced musculoskeletal sonographer. After repair, a traditional B-mode ultrasound was conducted to ensure the repair remained intact at each appointment.

### Surgical Arthroscopic Rotator Cuff Repair Technique

Indications for rotator cuff repair were patient pain, full-thickness tears, or partial-thickness tears (>50% thickness) that were also deemed repairable on ultrasound. For all patients, arthroscopic rotator cuff repair employing the undersurface technique approach was used, as detailed elsewhere.^18^ Postoperatively, each patient was then fastened into an abduction sling with a small cushioning pillow to be worn daily for the first 6 weeks postoperatively (DonJoy). The postrepair rehabilitation protocol is shown in Appendix Table A1.

### Level of Work and Sport Measurements

In their preoperative appointments, patients were asked to fill out forms that included stating their present level of sport and work participation (no activity, light activity, moderate activity, and strenuous activity) and their own highest level of sports and work activity previously performed (hobby: nonorganized sport; club: organized team sport without regional ranking; national or professional sport) on a 4-point Likert scale. Subsequently, the patient's activity level in sports and work was self-reported on the same Likert scale at 8 days, 6 weeks, 12 weeks, 6 months, and 12 months postoperatively.

### Shear Wave Elastography Ultrasound Examination

After arthroscopic rotator cuff repair, SWEUS measurements were taken on seated patients with shoulders neutrally rotated at zero abduction. Patients faced the sonographer anteriorly, with elbows at 90^o^, hands supinated in laps, and arms extended 35^o^ from neutral. The transducer was guided by the greater tuberosity and acromion (as lateral and medial landmarks, respectively), providing a longitudinal *birds-beak* view of the supraspinatus tendon, specifically 2 mm posterior to the biceps at the tendon footprint-articular cartilage junction.

All SWEUS velocity values (0.05 m/s to 10 m/sec ) were obtained through measurements taken by a single experienced musculoskeletal sonographer (L.H.) using the Linear 9L4 transducer of a Siemens Acuson S3000 ultrasound machine (Siemens Medical Solutions). Both our sonographer (L.H.) and ultrasound system have been shown to be very reliable.^7^

SWEUS velocity values were generated at 3 points along the tendon-bone interface as a measure of stiffness and digitally recorded for each patient. These areas of interest along the supraspinatus tendon were chosen to avoid bias caused by suture material upon the stiffness of the measured region. The deltoid muscle (low velocity, soft) was measured superior to the supraspinatus tendon as a negative control, and the positive control of the humeral head (high velocity, stiff) was selected for their contrasting SWEUS velocity values.

**Figure 2.**
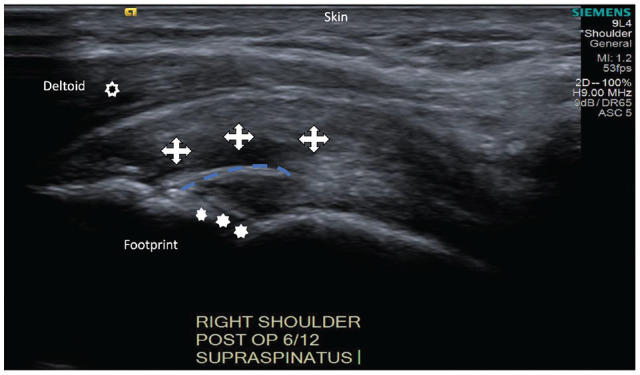
An ultrasound image showing 3 distinct supraspinatus tendon locations (crosses) along the tendon contour (blue line), humeral head (stars), and deltoid (hollow circle) measurements 3 mm apart.

Measured regions were taken by placing the sample box at the insertion site of the supraspinatus tendon into the greater tuberosity laterally (lateral point at the tendon-bone interface), 3 mm medial to the tendon-bone interface (mediolateral point), and 6 mm medial to the tendon-bone interface (medial point) (Figure 2). Orthogonal lines are depicted to account for the supraspinatus tendon curvature.

### Statistical Analysis

All data analysis was performed using SPSS Statistics 26 (IBM), with significance set at *P* < .05. Spearman rank correlation coefficients were used to examine the strength of correlation between velocity values obtained at each point along the supraspinatus tendon for each timepoint and patient work and sport activity levels at each postoperative timepoint. A 1-way analysis of variance with repeated measures was used to assess the tendon stiffness changes via SWEUS measurements at each of the 3 points along the supraspinatus tendon.

## Results

### Participant Characteristics

A total of 50 patients were recruited and 2 did not attend follow-up appointments, leaving a cohort of 48 patients (28 men, 20 women). Patients’ ages ranged between 24 and 85 years, with a median age of 57 years, and the mean tear size was 2.6 cm^2^ (range, 0.4-12 cm^2^). Six patients retore their tendons (3 at 6 weeks, 2 at 12 weeks, and 1 at 6 months) and were excluded from further SWEUS measurements due to tendon retraction rendering their tendons to be no longer assessable. SWEUS velocity scores were recorded at 8 days, 6 weeks, 12 weeks, 6 months, and 12 months.

As their highest level of preinjury sports activity, 40 patients reported having performed in *hobby sports*, 7 patients reported engaging in *club sports*, and 3 patients reported engaging in *national sports*. In the 8 days before their rotator cuff repair surgery, all patients had ceased sports activity, 28 patients ceased work entirely, 12 patients reported continuing *light activity*, 8 patients were still engaging in *moderate activity*, and 2 patients still engaged in *strenuous shoulder activity at work*.

### Control Measurements

The mean ± SD SWEUS velocity of the humeral head (as the positive control) was unchanged from 8 days (9.78±0.2m/s) to 12 months (9.86±0.1m/s) . The deltoid muscle (as the negative control) increased in SWEUS velocity from 3.4±0.9m/sec at 8 days to 4.1±1.0m/s at 12 months after repair (*P* = .003).

### Changes in the SWEUS Velocity of the Repaired Tendon

**Figure 3.**
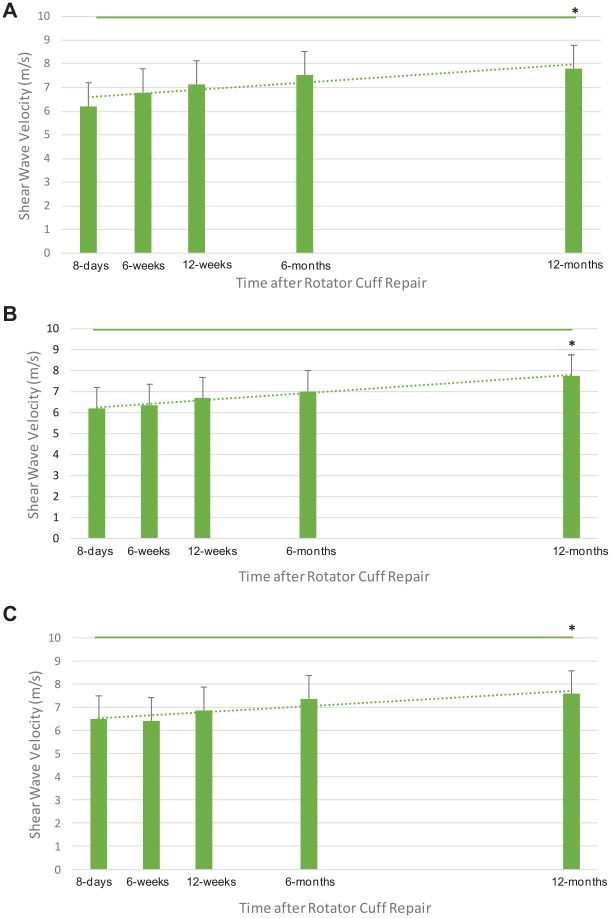
Shear wave velocity (mean ± SD) measurements of the healing supraspinatus tendon at the (A) lateral, (B) mediolateral (3 mm medial), and (C) medial (6 mm medial) points of the supraspinatus tendon along its insertion site after arthroscopic rotator cuff repair (n = 42). *P < .05 using 1-way analysis of variance. The dotted line denotes a linear trendline.

The SWEUS stiffness of the repaired supraspinatus tendon was found to improve in all 3 measured locations with time after rotator cuff repair, with a mean overall increase in elastography stiffness of 22% from 8 days to 12 months (P=.0001) (Figure 3).

At the lateral point of the arthroscopically repaired supraspinatus tendon, SWEUS velocity increased from 6.2±0.2m/s at 8 days to 7.8±0.3m/s at 12 months after repair ( P=0.0001 ). At the mediolateral point, SWEUS velocity increased from 6.2±0.2m/s at 8 days to 7.8±0.3m/s at 12 months after repair ( P=.001 ). Medially, SWEUS velocity increased from 6.5±0.2m/s at 8 days to 7.6±0.3m/s at 12 months postrepair (P=.03) . There were significant differences in the SWEUS-measured stiffness of the repaired supraspinatus tendon at all 3 measured locations (P=.0001) across all postoperative timepoints (P=.0001) , confirmed by a 3-way analysis of variance.

### Postoperative Supraspinatus Tendon Elastography Stiffness and Return to Work

**Figure 4.**
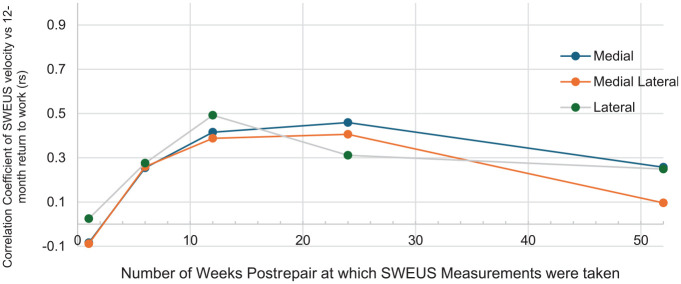
Timepoint of supraspinatus elastographic stiffness measurement versus correlation to return to work at 12 months postrepair. SWEU, shear wave elastography ultrasound.

A patient's return to work 12 months after the repair was most strongly associated with elastography-measured supraspinatus tendon stiffness 12 weeks after rotator cuff repair ( r=0.43;P<.01) , when averaged across the 3 measured points of the tendon (Figure 4).

**Figure 5.**
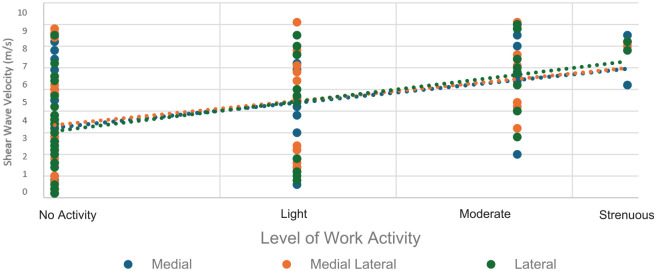
Supraspinatus elastographic stiffness measured at 12 weeks versus return to work activity at 12 months. SWEU, shear wave elastography ultrasound.

Tendon stiffness 12 weeks after repair was moderately associated with return to work at 12 months at all 3 measurement points of the tendon (medial: *r* = 0.42, *P* = .004; mediolateral: *r* = 0.39, *P* = .006; lateral: *r* = 0.49, *P* = .001) (Figure 5).

Tendon stiffness at 6 weeks (medial: *r* = 0.25, *P* = .046; mediolateral: *r* = 0.26, *P* = .042; lateral: *r* = 0.28, *P* = .03) and 6 months (medial: *r* = 0.46, *P* = .003; mediolateral: *r* = 0.41, *P* = .004; lateral: *r* = 0.31, *P* = .02) after repair were also significantly associated with return to work at 12 months.

### Postoperative Supraspinatus Tendon Elastography Stiffness and Return to Sports

**Figure 6.**
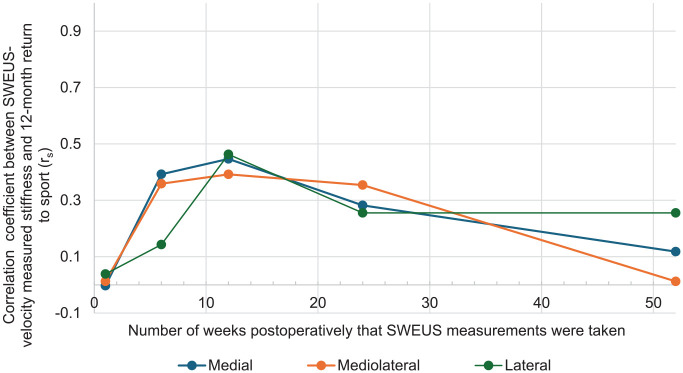
Timepoint of supraspinatus elastographic stiffness measurement versus correlation to return to sports at 12 months postrepair. SWEU, shear wave elastography ultrasound.

Patient return to sports at 12 months postrepair was most strongly associated with the 12-week elastography stiffness of the supraspinatus tendon at all 3 measured points (medial: *r* = 0.45, *P* = .002; mediolateral: *r* = 0.39, *P* = .006; lateral *r* = 0.46, *P* = .001) (Figure 6).

**Figure 7.**
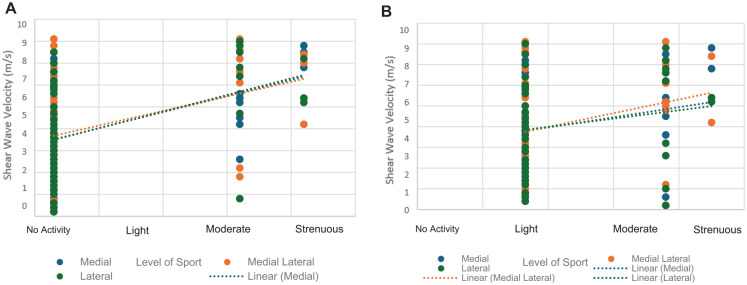
Elastographic supraspinatus tendon stiffness at 12 weeks versus return to sports at (A) 12 months and (B) 6 months.

Tendon stiffness at 12 weeks was also associated with the level of sports returned to 6 months after repair (*r* = 0.29; *P* = .004) (Figure 7).

**Figure 8.**
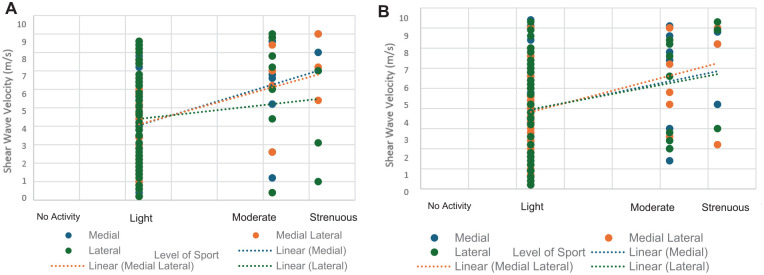
Elastographic supraspinatus tendon stiffness at (A) 6 weeks and (B) 6 months versus return to sports at 12 months.

Tendon stiffness at 6 weeks (medial: *r* = 0.39, *P* = .008; mediolateral: *r* = 0.36, *P* = .02) and 6 months (medial: *r* = 0.28, *P* = .04; mediolateral: *r* = 0.35, *P* = .02) were also significantly associated with return to sports at 12 months (Figure 8).

## Discussion

The major finding of the present study was that the stiffness of the repaired supraspinatus tendon, as assessed by SWEUS, is moderately associated with increased patient-reported return to work and sports at 12 months after repair. In measuring patients’ return to work and sports, the stiffness of the repaired supraspinatus tendon at 12 weeks postoperatively was most strongly associated with 12-month work and sports activity levels. The repaired supraspinatus tendons that were stiffest at 12 and 24 weeks postoperatively were most strongly associated with patient return to work at 12 months, while the stiffest tendons at 6 and 12 weeks were most strongly associated with return to sports after 12 months. Overall, these findings of stiffer healing tendons being associated with improved return to activity highlight the capacity for SWEUS to help inform clinicians and patients about the prospect of a return to work and sports 12 months postrepair after only 12 weeks.

Our data demonstrate moderate correlations between the elastography stiffness of repaired supraspinatus tendons at 12 weeks and the level of both sport and work to which patients return 12 months after rotator cuff repair (r=0.49andr=0.46, respectively). While no literature to our knowledge has evaluated the relationship between supraspinatus tendon stiffness and functional shoulder outcomes, there have been several other studies in humans using SWEUS to assess tendon healing.^13^ Itoigawa et al^11^ evaluated 60 patients from 1 week to 6 months after repair and found that the stiffness of the supraspinatus tendon decreased at the anteromedial anchor after double row repair—albeit with a different ultrasound machine (Aixplorer System) and without reporting the decreased velocity values. Nocera et al^14^ used SWEUS to evaluate the postrepair stiffness of supraspinatus tendons in 12 patients and found an initial decrease in stiffness at 3 months (r=−0.73;P=.005) and noted a trend of improved stiffness at 6 months postoperatively.

SWEUS has been shown to be a reliable tool for diagnosing rotator cuff tendinopathy.^11^ At our institution, Hackett et al^7^ examined the reliability of SWEUS in assessing supraspinatus tendons in 10 normal and 10 tendinopathic participants using virtual touch imaging quantification of the Siemens ACUSON S3000 ultrasound system. They found SWEUS velocity measurements to exhibit excellent intraobserver (ICC, 0.96) and moderate interobserver reliability^7^ (ICC, 0.45), similar to study findings by Baumer et al^3^ (ICC, 0.87). Hou et al^9^ also evaluated the association of SWEUS velocity to the degree of supraspinatus tendinopathy, finding the morphological grade of supraspinatus tendinopathy on B-mode ultrasound to be associated with decreased supraspinatus tendon stiffness (*P* < .003), demonstrating the capacity of SWEUS to identify tendons with decreased tensile properties.

We observed increasing SWEUS velocity in all 3 measured points of the repaired supraspinatus tendon from 8 days to 12 months, reflecting improved material properties of the tendon. However, similar to the findings of studies by Hou et al^9^ and Taljanovic et al,^19^ the repaired tendons in our study never reached the stiffness of normal tendons even at 12 months. The improved SWEUS velocity of the healing supraspinatus tendon may result from repair-phase tendon remodeling. In a study of 65 rotator cuff repairs, Yoo et al^20^ found a complete restoration of normal fibrillar pattern on ultrasound in 85% of repaired tendons at 6 months, reduced surface irregularity in 45%, and a loss of hypoechoic texture in 70%. Thus, our data support the hypothesis that the mechanical stiffness of the rotator cuff tendon is increased with an improved fibrillar pattern, surface regularity, and tendon texture. The observed increase in stiffness of repaired supraspinatus tendons in our study is also consistent with the findings of a study in 60 rats by Galatz et al,^5^ in which, a 66% improvement in supraspinatus tendon stiffness was observed up to 56 days after rotator cuff repair when testing the tendon load to failure.

### Strengths and Limitations

Our study strengths included the use of a single, experienced musculoskeletal sonographer whose intrauser reliability has been shown to be high^7^ and who conducted all shear wave ultrasound assessments, while the senior author (G.C.M.) performed and graded each rotator cuff repair. In addition, our study used bone for a positive control, muscle for a negative control, and a standardized sample box window size, which improved measurement reliability.^17^

Some weaknesses in our study design should be considered. The use of a Likert scale in patients describing their level of activity in work and sports participation could be limited because of the discrepancies in each patient's subjective rating of their activity. In addition, the data from this study did not investigate the impact of handedness or preoperative muscle quality on the identified associations, although these parameters have not been identified to correlate with SWEUS velocity stiffness.^13,16^ The use of a single surgeon, sonographer, and ultrasound machine reduced our study's external validity and applicability to those using alternative repair, intraoperative ranking, and velocity measurement techniques.^6^

## Conclusion

The data support the hypothesis that improved supraspinatus tendon stiffness at 12 weeks after repair is associated with patients being more likely to return to greater levels of activity in sports and work after 12 months. SWEU was also able to show that the supraspinatus tendon, as it heals, becomes stiffer over time after repair and stiffens faster laterally than it does medially.

## Appendices

**Appendix Table A1:** Rotator Cuff Repair Rehabilitation Protocol

Postoperative Timepoint	Rehabilitation Protocol
8 days	• No weighted activity
6 weeks	• Removal of sling • Light lifting between 1 and 2 kg below chest level • No overhead activities
12 weeks	• Light lifting between 2 and 5 kg • Light unweighted overhead activity
6 months	• Return to full activity
12 months	• Continue full activity
